# 3-Benzyl-1-{[3-(4-chloro­phen­yl)isoxazol-5-yl]meth­yl}-1*H*-benzimidazol-2(3*H*)-one

**DOI:** 10.1107/S160053681302299X

**Published:** 2013-08-17

**Authors:** Youssef Kandri Rodi, Amal Haoudi, Frédéric Capet, Christian Rolando, Lahcen El Ammari

**Affiliations:** a Laboratoire de Chimie Organique Appliquée, Université Sidi Mohamed Ben Abdallah, Faculté des Sciences et Techniques, Route d’Immouzzer, BP 2202 Fès, Morocco; b Unité de Catalyse et de Chimie du Solide (UCCS), UMR 8181, Ecole Nationale Supérieure de Chimie de Lille, France; c USR 3290 Miniaturisation pour l’Analyse, la Synthèse et la Protéomique, 59655 Villeneuve d’Ascq Cedex, Université Lille 1, France; d Laboratoire de Chimie du Solide Appliquée, Faculté des Sciences, Université Mohammed V-Agdal, Avenue Ibn Battouta, BP 1014, Rabat, Morocco

## Abstract

In the title compound, C_24_H_18_ClN_3_O_2_, the benzimidazole plane is nearly perpendicular to the phenyl ring and to the isoxazole ring, making dihedral angles of 75.95 (7) and 73.04 (8)°, respectively, but the two residues point in opposite directions with respect to the benzimidazole plane. The dihedral angle between the chloro­phenyl and isoxazole rings is 7.95 (8)°. In the crystal, mol­ecules are linked by pairs of C—H⋯O hydrogen bonds into inversion dimers.

## Related literature

For the biological activity of isoxazoline derivatives, see: Sakuma *et al.* (2011 ▶); Hu *et al.* (2010 ▶); Wang *et al.* (2010 ▶). For benzimidazol-2-one derivatives, see: Belaziz *et al.* (2012 ▶).
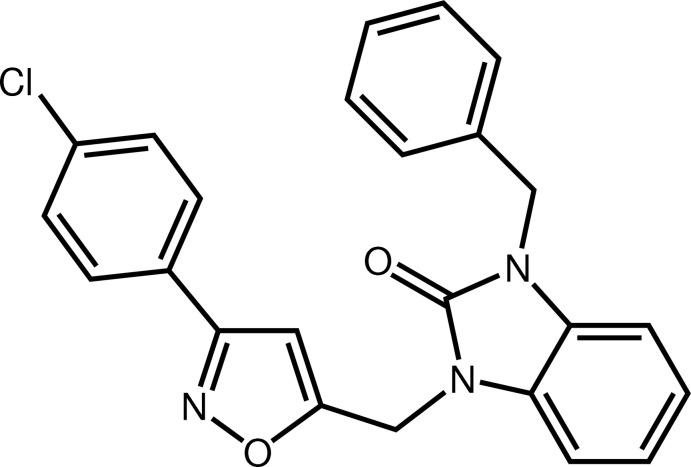


## Experimental

### 

#### Crystal data

C_24_H_18_ClN_3_O_2_
*M* *_r_* = 415.86
Triclinic, 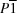

*a* = 8.5427 (2) Å
*b* = 9.8290 (2) Å
*c* = 13.2658 (3) Å
α = 81.133 (1)°
β = 78.763 (1)°
γ = 64.343 (1)°
*V* = 981.73 (4) Å^3^
*Z* = 2
Mo *K*α radiation
μ = 0.22 mm^−1^
*T* = 296 K
0.60 × 0.39 × 0.13 mm

#### Data collection

Bruker APEXII CCD diffractometer
Absorption correction: multi-scan (*SADABS*; Bruker, 2009 ▶) *T*_min_ = 0.688, *T*_max_ = 0.746
70806 measured reflections
5994 independent reflections
4622 reflections with *I* > 2σ(*I*)
*R*_int_ = 0.029

#### Refinement

*R*[*F* ^2^ > 2σ(*F* ^2^)] = 0.045
*wR*(*F* ^2^) = 0.143
*S* = 1.03
5994 reflections
271 parameters
H-atom parameters constrained
Δρ_max_ = 0.55 e Å^−3^
Δρ_min_ = −0.35 e Å^−3^

### 

Data collection: *APEX2* (Bruker, 2009 ▶); cell refinement: *APEX2* and *SAINT-Plus* (Bruker, 2009 ▶); data reduction: *SAINT-Plus*; program(s) used to solve structure: *SHELXS97* (Sheldrick, 2008 ▶); program(s) used to refine structure: *SHELXL97* (Sheldrick, 2008 ▶); molecular graphics: *ORTEP-3 for Windows* (Farrugia, 2012 ▶); software used to prepare material for publication: *PLATON* (Spek, 2009 ▶) and *publCIF* (Westrip, 2010 ▶).

## Supplementary Material

Crystal structure: contains datablock(s) I. DOI: 10.1107/S160053681302299X/bt6928sup1.cif

Structure factors: contains datablock(s) I. DOI: 10.1107/S160053681302299X/bt6928Isup2.hkl

Supplementary material file. DOI: 10.1107/S160053681302299X/bt6928Isup3.cml

Additional supplementary materials:  crystallographic information; 3D view; checkCIF report

## Figures and Tables

**Table 1:** Hydrogen-bond geometry (Å, °)

*D*—H⋯*A*	*D*—H	H⋯*A*	*D*⋯*A*	*D*—H⋯*A*
C20—H20⋯O2^i^	0.93	2.53	3.1949 (19)	129

Symmetry code: (i) 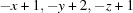.

**Fig. 1.**
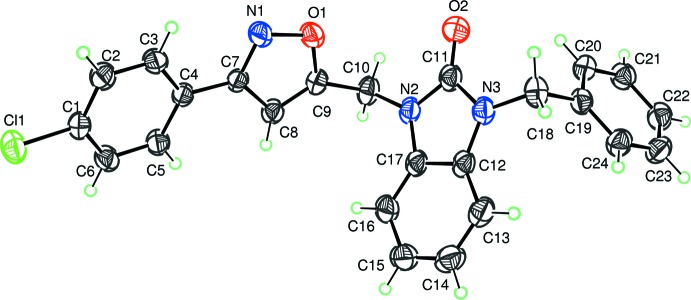
: Molecular plot the title compound with the atom-labelling scheme. Displacement ellipsoids are drawn at the 50% probability level. H atoms are represented as small circles.

**Fig. 2.**
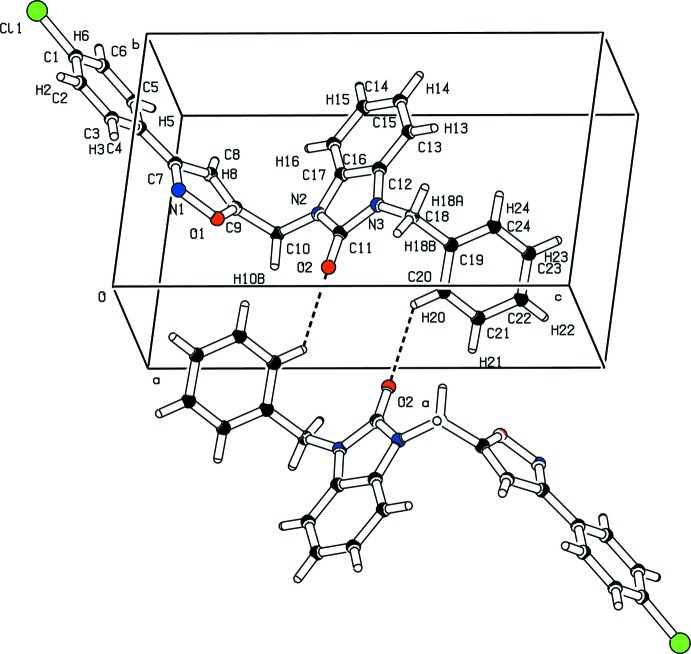
: Intermolecular interactions in the title compound building a dimers. Hydrogen bonds are shown as dashed lines.

## supplementary crystallographic information

### 1. Comment

Isoxazoline derivatives have attracted considerable attention and are found in many important bioactive heterocycles. These pharmacophores play an important role in medicinal (Sakuma *et al.*, 2011, Wang *et al.*, 2010) and agrochemical industry (Hu *et al.*, 2010). The integration of isoxazoline moiety in benzimidazole scaffolds may lead to new hybrid molecules containing two pharmacophores in the same molecule with broad biological activity profiles.

As a continuation of our research work devoted to the development of substituted benzimidazol-2-one derivatives (Belaziz *et al.*, 2012), we report in this paper the synthesis of a new isoxazolinylmethyl benzimidazole derivatives. The title compound was obtained by using *p*-chlorobenzaldoxime to react with the 1-allyl-3-benzyl-benzimidazole in 1,3-dipolar cycloaddition.

The title compound is build up from a fused five- and six-membered rings linked, on opposite sides, to a benzyl residue and to a chlorobenzyl- dihydroisoxazole residue (Fig. 1). The benzimidazole plane makes dihedral angles of 75.95 (7) ° and 73.04 (8) ° with the phenyl ring and the isoxazole ring, respectively. The dihedral angle between the chlorophenyl ring and the isoxazole ring is of 7.95 (8) ° while that between the two aromatic six-membered rings is 68.48 (8)°.

In the crystal, the molecules are linked by C–H···O hydrogen bonds to centrosymmetric dimers (Fig. 2 and Table 2).

### 2. Experimental

To 1-allylbenzimidozol-2-one (0.40 g, 2.3 mmol), potassium carbonate (0.63 g, 4,55 mmol) and tetra-n-butylammonium bromide (0.07 g, 0.22 mmol) in DMF (15 ml) was added benzyl chloride (0.32 g, 2.53 mmol). Stirring was continued at room temperature for 6 h. The salt was removed by filtration and the filtrate concentrated under reduced pressure. The residue was separated by chromatography on a column of silica gel with ethyl acetate/ hexane (1/2) as eluent. The compound was recrystallized from hexane/acetate to give colorless crystals. To the obtained compound (1-allyl-3-benzyl-benzimidazol-2-one) (0.20 g, 0.76 mmol) was added *p*-chlorobenzaldoxime (0.15 g, 1 mmol) in chloroform (10 ml) and 4% solution of sodium hypochlorite solution (commercial bleach) (4 ml) at 0°C. Stirring was continued for 6 h. The organic layer was dried and the solvent evaporated under reduced pressure. The residue was then purified by column chromatography on silica gel by using a mixture of hexane and ethyl acetate (1/1) as eluent. Colourless crystals were isolated when the solvent was allowed to evaporate (Yield: 68%; mp: 467 K).

### 3. Refinement

All H atoms could be located in a difference Fourier map. Nevertheless, they were placed in calculated positions with C—H = 0.93 Å (aromatic), and C—H = 0.97 Å (methylene) and refined as riding on their parent atoms with *U*_iso_(H) = 1.2 *U*_eq_ (C).

### Crystal data

**Table:**

C_24_H_18_ClN_3_O_2_	*Z* = 2
*M**_r_* = 415.86	*F*(000) = 432
Triclinic, *P*1	*D*_x_ = 1.407 Mg m^−^^3^
Hall symbol: -P 1	Melting point: 467 K
*a* = 8.5427 (2) Å	Mo *K*α radiation, λ = 0.71073 Å
*b* = 9.8290 (2) Å	Cell parameters from 9881 reflections
*c* = 13.2658 (3) Å	θ = 2.7–29.9°
α = 81.133 (1)°	µ = 0.22 mm^−^^1^
β = 78.763 (1)°	*T* = 296 K
γ = 64.343 (1)°	Prism, colourless
*V* = 981.73 (4) Å^3^	0.60 × 0.39 × 0.13 mm

### Data collection

**Table:**

Bruker APEXII CCD diffractometer	5994 independent reflections
Radiation source: fine-focus sealed tube	4622 reflections with *I* > 2σ(*I*)
Graphite monochromator	*R*_int_ = 0.029
φ and ω scans	θ_max_ = 30.6°, θ_min_ = 2.3°
Absorption correction: multi-scan (*SADABS*; Bruker, 2009)	*h* = −12→12
*T*_min_ = 0.688, *T*_max_ = 0.746	*k* = −14→14
70806 measured reflections	*l* = −18→18

### Refinement

**Table:**

Refinement on *F*^2^	Primary atom site location: structure-invariant direct methods
Least-squares matrix: full	Secondary atom site location: difference Fourier map
*R*[*F*^2^ > 2σ(*F*^2^)] = 0.045	Hydrogen site location: inferred from neighbouring sites
*wR*(*F*^2^) = 0.143	H-atom parameters constrained
*S* = 1.03	*w* = 1/[σ^2^(*F*_o_^2^) + (0.0745*P*)^2^ + 0.2335*P*] where *P* = (*F*_o_^2^ + 2*F*_c_^2^)/3
5994 reflections	(Δ/σ)_max_ = 0.001
271 parameters	Δρ_max_ = 0.55 e Å^−^^3^
0 restraints	Δρ_min_ = −0.35 e Å^−^^3^

### Special details

**Table:**

Geometry. All s.u.'s (except the s.u. in the dihedral angle between two l.s. planes) are estimated using the full covariance matrix. The cell s.u.'s are taken into account individually in the estimation of s.u.'s in distances, angles and torsion angles; correlations between s.u.'s in cell parameters are only used when they are defined by crystal symmetry. An approximate (isotropic) treatment of cell s.u.'s is used for estimating s.u.'s involving l.s. planes.
Refinement. Refinement of *F*^2^ against all reflections. The weighted *R*-factor *wR* and goodness of fit *S* are based on *F*^2^, conventional *R*-factors *R* are based on *F*, with *F* set to zero for negative *F*^2^. The threshold expression of *F*^2^ > σ(*F*^2^) is used only for calculating *R*-factors(gt) *etc*. and is not relevant to the choice of reflections for refinement. *R*-factors based on *F*^2^ are statistically about twice as large as those based on *F*, and *R*- factors based on all data will be even larger.

### Fractional atomic coordinates and isotropic or equivalent isotropic displacement parameters (Å^2^)

**Table:**

	*x*	*y*	*z*	*U*_iso_*/*U*_eq_	
C1	0.96270 (18)	0.07935 (15)	1.15305 (10)	0.0408 (3)	
C2	1.0111 (2)	0.19835 (18)	1.13183 (12)	0.0491 (3)	
H2	1.0951	0.2011	1.1658	0.059*	
C3	0.9335 (2)	0.31396 (17)	1.05931 (11)	0.0466 (3)	
H3	0.9659	0.3947	1.0446	0.056*	
C4	0.80752 (16)	0.31087 (14)	1.00813 (9)	0.0362 (2)	
C5	0.75890 (19)	0.19111 (17)	1.03279 (11)	0.0445 (3)	
H5	0.6728	0.1890	1.0004	0.053*	
C6	0.8363 (2)	0.07437 (17)	1.10491 (12)	0.0468 (3)	
H6	0.8035	−0.0061	1.1206	0.056*	
C7	0.73329 (17)	0.42911 (14)	0.92671 (9)	0.0362 (2)	
C8	0.62184 (18)	0.43492 (16)	0.85805 (10)	0.0422 (3)	
H8	0.5746	0.3661	0.8565	0.051*	
C9	0.59949 (17)	0.56105 (15)	0.79592 (10)	0.0400 (3)	
C10	0.50513 (19)	0.63221 (18)	0.70493 (11)	0.0480 (3)	
H10A	0.4119	0.5997	0.7084	0.058*	
H10B	0.4520	0.7414	0.7069	0.058*	
C11	0.6890 (2)	0.68917 (17)	0.54875 (11)	0.0461 (3)	
C12	0.79140 (18)	0.47023 (16)	0.47125 (10)	0.0436 (3)	
C13	0.8769 (2)	0.35197 (19)	0.40860 (12)	0.0562 (4)	
H13	0.9492	0.3597	0.3478	0.067*	
C14	0.8507 (3)	0.2209 (2)	0.43976 (15)	0.0669 (5)	
H14	0.9087	0.1387	0.3996	0.080*	
C15	0.7409 (3)	0.20923 (19)	0.52889 (14)	0.0628 (4)	
H15	0.7239	0.1208	0.5465	0.075*	
C16	0.6556 (2)	0.32735 (18)	0.59249 (12)	0.0522 (3)	
H16	0.5825	0.3196	0.6529	0.063*	
C17	0.68346 (18)	0.45689 (16)	0.56272 (10)	0.0423 (3)	
C18	0.8719 (2)	0.68311 (19)	0.37749 (12)	0.0511 (3)	
H18A	0.9817	0.6069	0.3471	0.061*	
H18B	0.8978	0.7582	0.4019	0.061*	
C19	0.75256 (18)	0.75806 (16)	0.29623 (10)	0.0425 (3)	
C20	0.6070 (2)	0.89424 (17)	0.31390 (11)	0.0463 (3)	
H20	0.5858	0.9388	0.3750	0.056*	
C21	0.4936 (2)	0.96400 (18)	0.24177 (12)	0.0511 (3)	
H21	0.3960	1.0546	0.2547	0.061*	
C22	0.5253 (2)	0.8988 (2)	0.15014 (12)	0.0541 (4)	
H22	0.4493	0.9457	0.1014	0.065*	
C23	0.6691 (2)	0.7652 (2)	0.13163 (12)	0.0570 (4)	
H23	0.6909	0.7219	0.0699	0.068*	
C24	0.7823 (2)	0.69410 (19)	0.20462 (12)	0.0519 (3)	
H24	0.8788	0.6029	0.1918	0.062*	
Cl1	1.06618 (6)	−0.06917 (5)	1.24013 (3)	0.05818 (13)	
N1	0.7736 (2)	0.54512 (15)	0.90688 (11)	0.0558 (3)	
N2	0.62261 (16)	0.59181 (13)	0.60857 (8)	0.0439 (3)	
N3	0.79299 (16)	0.61217 (14)	0.46473 (9)	0.0460 (3)	
O1	0.68832 (17)	0.63109 (13)	0.82270 (9)	0.0573 (3)	
O2	0.66140 (18)	0.81529 (13)	0.56718 (9)	0.0607 (3)	

### Atomic displacement parameters (Å^2^)

**Table:**

	*U*^11^	*U*^22^	*U*^33^	*U*^12^	*U*^13^	*U*^23^
C1	0.0437 (6)	0.0438 (6)	0.0331 (6)	−0.0174 (5)	−0.0066 (5)	0.0017 (5)
C2	0.0552 (8)	0.0538 (8)	0.0485 (8)	−0.0288 (7)	−0.0227 (6)	0.0051 (6)
C3	0.0585 (8)	0.0477 (7)	0.0469 (7)	−0.0324 (6)	−0.0205 (6)	0.0059 (6)
C4	0.0397 (6)	0.0411 (6)	0.0304 (5)	−0.0194 (5)	−0.0054 (4)	−0.0021 (4)
C5	0.0482 (7)	0.0530 (7)	0.0425 (7)	−0.0304 (6)	−0.0137 (5)	0.0045 (6)
C6	0.0534 (8)	0.0501 (7)	0.0465 (7)	−0.0320 (6)	−0.0106 (6)	0.0061 (6)
C7	0.0397 (6)	0.0404 (6)	0.0302 (5)	−0.0181 (5)	−0.0046 (4)	−0.0033 (4)
C8	0.0461 (7)	0.0499 (7)	0.0370 (6)	−0.0251 (6)	−0.0112 (5)	0.0008 (5)
C9	0.0389 (6)	0.0455 (7)	0.0324 (6)	−0.0146 (5)	−0.0045 (5)	−0.0037 (5)
C10	0.0426 (7)	0.0542 (8)	0.0364 (6)	−0.0096 (6)	−0.0093 (5)	−0.0002 (6)
C11	0.0496 (7)	0.0452 (7)	0.0376 (6)	−0.0130 (6)	−0.0134 (5)	0.0017 (5)
C12	0.0442 (7)	0.0445 (7)	0.0364 (6)	−0.0102 (5)	−0.0162 (5)	0.0010 (5)
C13	0.0591 (9)	0.0556 (9)	0.0421 (7)	−0.0096 (7)	−0.0122 (6)	−0.0073 (6)
C14	0.0808 (12)	0.0497 (9)	0.0620 (10)	−0.0106 (8)	−0.0250 (9)	−0.0130 (8)
C15	0.0821 (12)	0.0489 (8)	0.0619 (10)	−0.0252 (8)	−0.0306 (9)	0.0021 (7)
C16	0.0599 (9)	0.0520 (8)	0.0465 (8)	−0.0227 (7)	−0.0213 (7)	0.0069 (6)
C17	0.0436 (6)	0.0438 (7)	0.0355 (6)	−0.0116 (5)	−0.0174 (5)	0.0027 (5)
C18	0.0454 (7)	0.0613 (9)	0.0436 (7)	−0.0219 (7)	−0.0084 (6)	0.0060 (6)
C19	0.0439 (7)	0.0489 (7)	0.0364 (6)	−0.0232 (6)	−0.0040 (5)	0.0018 (5)
C20	0.0536 (8)	0.0479 (7)	0.0382 (6)	−0.0217 (6)	−0.0083 (6)	−0.0015 (5)
C21	0.0522 (8)	0.0506 (8)	0.0506 (8)	−0.0212 (6)	−0.0133 (6)	0.0026 (6)
C22	0.0599 (9)	0.0698 (10)	0.0467 (8)	−0.0390 (8)	−0.0180 (7)	0.0053 (7)
C23	0.0686 (10)	0.0752 (11)	0.0416 (7)	−0.0425 (9)	−0.0045 (7)	−0.0115 (7)
C24	0.0528 (8)	0.0564 (8)	0.0448 (7)	−0.0226 (7)	0.0005 (6)	−0.0091 (6)
Cl1	0.0641 (2)	0.0540 (2)	0.0521 (2)	−0.02165 (18)	−0.01949 (17)	0.01336 (16)
N1	0.0801 (9)	0.0523 (7)	0.0520 (7)	−0.0390 (7)	−0.0331 (7)	0.0130 (6)
N2	0.0495 (6)	0.0440 (6)	0.0317 (5)	−0.0131 (5)	−0.0093 (4)	0.0009 (4)
N3	0.0494 (6)	0.0461 (6)	0.0368 (6)	−0.0158 (5)	−0.0077 (5)	0.0022 (5)
O1	0.0810 (8)	0.0494 (6)	0.0542 (6)	−0.0357 (6)	−0.0315 (6)	0.0140 (5)
O2	0.0779 (8)	0.0466 (6)	0.0534 (6)	−0.0220 (6)	−0.0090 (6)	−0.0044 (5)

### Geometric parameters (Å, º)

**Table:**

C1—C2	1.375 (2)	C12—C17	1.400 (2)
C1—C6	1.378 (2)	C13—C14	1.389 (3)
C1—Cl1	1.7342 (13)	C13—H13	0.9300
C2—C3	1.384 (2)	C14—C15	1.382 (3)
C2—H2	0.9300	C14—H14	0.9300
C3—C4	1.3928 (18)	C15—C16	1.386 (2)
C3—H3	0.9300	C15—H15	0.9300
C4—C5	1.3845 (18)	C16—C17	1.380 (2)
C4—C7	1.4701 (17)	C16—H16	0.9300
C5—C6	1.386 (2)	C17—N2	1.3882 (18)
C5—H5	0.9300	C18—N3	1.4610 (19)
C6—H6	0.9300	C18—C19	1.509 (2)
C7—N1	1.3037 (18)	C18—H18A	0.9700
C7—C8	1.4192 (17)	C18—H18B	0.9700
C8—C9	1.3394 (19)	C19—C24	1.383 (2)
C8—H8	0.9300	C19—C20	1.391 (2)
C9—O1	1.3449 (18)	C20—C21	1.381 (2)
C9—C10	1.4922 (18)	C20—H20	0.9300
C10—N2	1.4527 (18)	C21—C22	1.386 (2)
C10—H10A	0.9700	C21—H21	0.9300
C10—H10B	0.9700	C22—C23	1.371 (3)
C11—O2	1.2124 (19)	C22—H22	0.9300
C11—N3	1.3772 (19)	C23—C24	1.390 (2)
C11—N2	1.388 (2)	C23—H23	0.9300
C12—C13	1.379 (2)	C24—H24	0.9300
C12—N3	1.3902 (19)	N1—O1	1.4088 (16)
			
C2—C1—C6	121.31 (13)	C15—C14—H14	119.1
C2—C1—Cl1	119.23 (11)	C13—C14—H14	119.1
C6—C1—Cl1	119.44 (11)	C14—C15—C16	121.02 (17)
C1—C2—C3	119.24 (13)	C14—C15—H15	119.5
C1—C2—H2	120.4	C16—C15—H15	119.5
C3—C2—H2	120.4	C17—C16—C15	117.41 (16)
C2—C3—C4	120.78 (13)	C17—C16—H16	121.3
C2—C3—H3	119.6	C15—C16—H16	121.3
C4—C3—H3	119.6	C16—C17—N2	131.99 (14)
C5—C4—C3	118.59 (12)	C16—C17—C12	121.66 (14)
C5—C4—C7	120.80 (11)	N2—C17—C12	106.35 (13)
C3—C4—C7	120.56 (12)	N3—C18—C19	111.88 (12)
C4—C5—C6	121.11 (12)	N3—C18—H18A	109.2
C4—C5—H5	119.4	C19—C18—H18A	109.2
C6—C5—H5	119.4	N3—C18—H18B	109.2
C1—C6—C5	118.94 (13)	C19—C18—H18B	109.2
C1—C6—H6	120.5	H18A—C18—H18B	107.9
C5—C6—H6	120.5	C24—C19—C20	118.70 (13)
N1—C7—C8	110.87 (12)	C24—C19—C18	121.82 (14)
N1—C7—C4	120.56 (11)	C20—C19—C18	119.47 (13)
C8—C7—C4	128.52 (12)	C21—C20—C19	120.76 (14)
C9—C8—C7	105.03 (12)	C21—C20—H20	119.6
C9—C8—H8	127.5	C19—C20—H20	119.6
C7—C8—H8	127.5	C20—C21—C22	119.91 (15)
C8—C9—O1	109.84 (12)	C20—C21—H21	120.0
C8—C9—C10	133.67 (14)	C22—C21—H21	120.0
O1—C9—C10	116.44 (13)	C23—C22—C21	119.81 (15)
N2—C10—C9	111.65 (11)	C23—C22—H22	120.1
N2—C10—H10A	109.3	C21—C22—H22	120.1
C9—C10—H10A	109.3	C22—C23—C24	120.32 (14)
N2—C10—H10B	109.3	C22—C23—H23	119.8
C9—C10—H10B	109.3	C24—C23—H23	119.8
H10A—C10—H10B	108.0	C19—C24—C23	120.49 (15)
O2—C11—N3	127.39 (15)	C19—C24—H24	119.8
O2—C11—N2	127.01 (14)	C23—C24—H24	119.8
N3—C11—N2	105.60 (13)	C7—N1—O1	105.96 (11)
C13—C12—N3	132.02 (15)	C17—N2—C11	110.57 (12)
C13—C12—C17	120.70 (15)	C17—N2—C10	126.87 (13)
N3—C12—C17	107.24 (12)	C11—N2—C10	122.56 (13)
C12—C13—C14	117.41 (16)	C11—N3—C12	110.25 (12)
C12—C13—H13	121.3	C11—N3—C18	122.37 (13)
C14—C13—H13	121.3	C12—N3—C18	126.95 (13)
C15—C14—C13	121.78 (16)	C9—O1—N1	108.30 (11)
			
C6—C1—C2—C3	−1.2 (2)	C24—C19—C20—C21	0.4 (2)
Cl1—C1—C2—C3	177.46 (12)	C18—C19—C20—C21	−178.77 (14)
C1—C2—C3—C4	0.1 (2)	C19—C20—C21—C22	−0.6 (2)
C2—C3—C4—C5	1.3 (2)	C20—C21—C22—C23	0.1 (2)
C2—C3—C4—C7	−176.27 (13)	C21—C22—C23—C24	0.5 (3)
C3—C4—C5—C6	−1.6 (2)	C20—C19—C24—C23	0.3 (2)
C7—C4—C5—C6	175.95 (13)	C18—C19—C24—C23	179.41 (15)
C2—C1—C6—C5	0.9 (2)	C22—C23—C24—C19	−0.7 (3)
Cl1—C1—C6—C5	−177.76 (11)	C8—C7—N1—O1	−0.27 (17)
C4—C5—C6—C1	0.5 (2)	C4—C7—N1—O1	177.18 (12)
C5—C4—C7—N1	176.96 (14)	C16—C17—N2—C11	179.49 (14)
C3—C4—C7—N1	−5.6 (2)	C12—C17—N2—C11	0.00 (15)
C5—C4—C7—C8	−6.1 (2)	C16—C17—N2—C10	−0.9 (2)
C3—C4—C7—C8	171.39 (14)	C12—C17—N2—C10	179.61 (12)
N1—C7—C8—C9	0.37 (17)	O2—C11—N2—C17	−179.88 (14)
C4—C7—C8—C9	−176.82 (12)	N3—C11—N2—C17	−0.18 (15)
C7—C8—C9—O1	−0.32 (15)	O2—C11—N2—C10	0.5 (2)
C7—C8—C9—C10	177.14 (14)	N3—C11—N2—C10	−179.80 (12)
C8—C9—C10—N2	−96.06 (19)	C9—C10—N2—C17	78.91 (18)
O1—C9—C10—N2	81.27 (16)	C9—C10—N2—C11	−101.52 (15)
N3—C12—C13—C14	177.73 (15)	O2—C11—N3—C12	179.99 (15)
C17—C12—C13—C14	0.3 (2)	N2—C11—N3—C12	0.29 (15)
C12—C13—C14—C15	1.2 (3)	O2—C11—N3—C18	−7.1 (2)
C13—C14—C15—C16	−1.7 (3)	N2—C11—N3—C18	173.20 (12)
C14—C15—C16—C17	0.6 (2)	C13—C12—N3—C11	−178.00 (15)
C15—C16—C17—N2	−178.55 (14)	C17—C12—N3—C11	−0.30 (15)
C15—C16—C17—C12	0.9 (2)	C13—C12—N3—C18	9.5 (2)
C13—C12—C17—C16	−1.4 (2)	C17—C12—N3—C18	−172.80 (13)
N3—C12—C17—C16	−179.37 (12)	C19—C18—N3—C11	−89.12 (17)
C13—C12—C17—N2	178.19 (13)	C19—C18—N3—C12	82.55 (18)
N3—C12—C17—N2	0.18 (14)	C8—C9—O1—N1	0.17 (16)
N3—C18—C19—C24	−104.54 (17)	C10—C9—O1—N1	−177.78 (12)
N3—C18—C19—C20	74.59 (18)	C7—N1—O1—C9	0.07 (17)

### Hydrogen-bond geometry (Å, º)

**Table:**

*D*—H···*A*	*D*—H	H···*A*	*D*···*A*	*D*—H···*A*
C20—H20···O2^i^	0.93	2.53	3.1949 (19)	129

Symmetry code: (i) −*x*+1, −*y*+2, −*z*+1.
